# A Radiographic comparison of mandibular bone quality in pre- and post-menopausal women in Indian population

**DOI:** 10.4103/0972-124X.70833

**Published:** 2010

**Authors:** Jayashree A. Mudda, Monika Bajaj, Veena A. Patil

**Affiliations:** *Department of Periodontics, H.K.E Society’s S. N. Institute of Dental Sciences and Research, Gulbarga, Karnataka, India*

**Keywords:** Mandibular cortical index, mental index, osteoporosis, panoramic mandibular index

## Abstract

**Aim and Objectives:**

The main objective of the study is to assess mandibular bone changes in pre and postmenopausal women with chronic generalized periodontitis using different radiomorphometric indices, mandibular cortical index (MCI), mental index (MI), and panoramic mandibular index (PMI) in Indian population.

**Materials and Methods:**

Panoramic radiographs of 60 patients visiting the Department of Periodontology were taken and divided in two groups - pre and post-menopausal. Detailed medical and menopausal history was recorded for each patient. All the radiographs were assessed for PMI, MI and MCI and presence of periodontitis. The destructive periodontitis was assessed as distance from the cementoenamel junction to the alveolar crest greater than 2mm.

**Statistical Analysis:**

Student-*t* test was applied to compare mean values of MI and PMI. Intra and inter group comparison of MCI were made using chi-square test. Correlation of age and MI and PMI was found by Pearson’s correlation coefficient.

**Results:**

MCI, PMI and MI were related to the menopausal status. Patients with C3 category were seen only in post-menopausal group after 54 years of age. Higher mean values for both MI (*p*<0.05) and PMI (*p*<0.05) were observed in pre-menopausal group with statistically significant differences. MI showed negative correlation with age in both the groups; however PMI was positively correlated with age.

**Conclusions:**

Radiomorphometric indices could be used by general dentists after a little training to detect post-menopausal women at higher risk of osteoporosis.

## INTRODUCTION

In humans, the bone mass decreases with increasing age. Human bones decrease in density and increase in porosity beginning at approximately the third decade of life.[1] This decline in bone mass is accelerated in women after menopause[2] and the rate of bone loss has been reported to vary from 0.5 to 1% per year.[3] Thus menopause puts women at greater risk of osteoporosis. The bone mineral density (BMD) of mandible is shown to be affected by mineral status of the skeleton and also by general diseases that causes generalized bone loss.[4] Various studies have reported that decreased BMD affects the morphometric,[5–8] densitometric[9,10] and architectural properties[11–13] of mandibular bone. Horner *et al*,[14] in a study reported mandibular body to be most appropriate site for any planned assessment of validity of mandibular measurements as a predictor of general bone mass. Although bone densitometry is accepted as the gold standard in diagnosis of osteoporosis[15] and a large number of methods for assessment of bone loss have been proposed (dual photon absorptiometry (DPA), dual energy X-ray absorptiometry (DXA), single photon absorptiometry (SPA), quantitative computed tomography (QCT),[3] one of the simplest method in the dental evaluation of mandibular bone loss is dental panoramic radiograph (DPR).[16] Radiographic bone density can be assessed from simple radiographs in two main ways: by taking linear measurements (morpho -metric analysis) or by measuring optical density of bone and comparing it with a reference step wedge (densitometric analysis).[17,18] Morphometric analysis has been limited to cortical thickness measurements at various sites[17,18] and calculation of the panoramic mandibular index.[19,20] Previous studies have reported significant correlations between mandibular BMD measurement by DXA method and mandibular cortical thickness or panoramic mandibular index.[9]

Periodontitis is an inflammatory disease that leads to alveolar bone loss; severe osteoporosis could be suspected of being an aggravating factor in case of periodontal destruction.[2] Until now studies done have evaluated various radiomorphometric indices to determine the range of these indices in a defined population. Thus the aim of the study was to compare the quantity and quality of mandibular bone with the help of morphometric analysis in pre and post-menopausal women having chronic generalized periodontitis undergoing physiological bone changes using dental panoramic radiographs.

## MATERIALS AND METHODS

### Subject Selection

Sixty female patients, who had received a dental panoramic radiograph (DPR) as a part of their dental treatment, were selected randomly from the Department of Periodontology. All the patients were informed about the study and informed consent was signed by each patient. A detailed medical history and menopausal history (if present) was recorded for each patient. All the patients had chronic generalized periodontitis. Presence of periodontitis was assessed both clinically and radiographically on their panoramic radiographs[21]. The inclusion criteria were:

1. Post-menopausal women not undergoing hormone replacement therapy or calcium supplement therapy.
2. Patients who had not undergone hysterectomy or oophorectomy.
3. Patients free from systemic diseases affecting BMD, bone lesions, fracture, or deformity or previous mandibular surgery.
4. Patients having bone loss of more than 2mm from CEJ at more than 33% of sites.
5. Patients with periodontal pocket depth of ≥5 mm at >33% of sites.

Patients on medication, affecting the BMD [steroids, anticonvulsants, excessive thyroxine doses], were excluded from the study.

### Radiographic examination

All panoramic images were made using the panoramic machine (Trophy Radiologie, Type OPX/105, France) at 110kv and 100mA utilizing Kodak films. All the images were developed manually and digitized after drying using a professional Epson scanner at 300dpi.

### Linear radiomorphometric measurement

All the linear measurements were made by using computerized dental software Dental Eye^™^3 by a single observer, a post graduate student in department of peridontics under the guidance of senior faculty.

### Following indices were measured on DPRs

1. MCI: This is a classification of the appearance of the lower border cortex of the mandible distal to the mental foramina, as viewed on panoramic radiographs as described by Klemetti *et al*.[18]
  On a three-point scale
  C1: the endosteal margin of the cortex is even and sharp on both sides.
  C2: the endosteal margin shows semilunar defects (lacunar resorption) or seems to form endosteal cortical residues (one to three layers) on one or both sides.
  C3: the cortical layer forms heavy endosteal residues and is clearly porous.
2. Panoramic mandibular index (PMI): Ratio of the mandibular cortical thickness measured on the line perpendicular to the bottom of the mandible, at the middle of the mental foramen, by the distance between the inferior mandibular cortex and the bottom of the mandible[19] (normal value:≥0.3).
3. Mental index (MI): Mandibular cortical thickness measured on the line perpendicular to the bottom of the mandible at the center of the mental foramen (normal value≥3.1 mm).[22]

## RESULTS

Mean age in the pre-menopausal (pre-M) group was 32.43±8.22 years and 54.26±10.14 years in post-menopausal (post-M) group. All the measurements were made for left and right sides of the mandible and mean of both sides was used for further calculations.

Mean MI was 4.99 in pre-menopausal and 4.46 in postmenopausal group (*P*<0.05). Mean of PMI was 0.41 in pre-M and 0.36 in post-M group (*P*<0.05). Student *t*-test was applied to compare mean MI and PMI values in both the groups and statistically significant difference was found between two groups [Table 1].

**Table 1:** Inter-group analysis of MI and PMI

	Pre-M Mean±S.D*	Post-M Mean±S.D	*P*-value
MI (mm)	4.99±0.75	4.46±1.09	<0.05
PMI	0.41±0.04	0.36±0.10	<0.05

* Standard deviation

Pearson’s correlation coefficient was used to correlate MI and PMI with age and age was found to be correlated with MI and PMI in both patients groups. MI showed a negative relation with age but PMI was positively correlated [Table 2]. Post-M group was sub-divided into two groups based on age and a negative correlation was seen with age after the age of 60 years [Table 3].

**Table 2:** Correlation of MI and PMI with age

	Correlation coefficient (r), MI	Correlation coefficient (r), PMI
Pre-M	-0.10	0.16
Post-M	-0.19	0.057

**Table 3:** Correlation of PMI with age in sub-groups of post-M group

	45-60 years	60-80 years
Correlation coefficient (r)	0.23	-0.98

MCI is a qualitative index. On intra group analysis with Chi-square test, no association was found between MCI grade on right and left side and menopausal status in both the groups [Table 4]. But when compared between the groups MCI was associated with the menopausal status on both the right and left side [Table 5].

**Table 4:** Intra-group comparison of MCI

	Pre-M		Post-M		
	C1	C2	C1	C2	C3
Right side	29	1	3	24	3
Left side	29	1	4	23	3
	*(X_2d.f_=0, p>0.05)*		*(X_2d.f_=0.16, p>0.05)*		

**Table 5:** Inter-group comparison of MCI

	Right side			Left side		
	C1	C2	C3	C1	C2	C3
Pre-M	29	01	00	29	01	00
Post-M	03	24	03	4	23	3
	*(X_2d.f_ =45.29, p<0.001)*			*(X_2d.f_=42.11, p<0.001)*		

Chi-square test was applied to find the association between periodontitis with MCI and no association was found between MCI status and periodontitis on either right or left side in both the groups [Tables 6 and 7].

**Table 6:** Association of periodontitis and MCI in Pre-M group (intra-group analysis)

Periodontitis	Pre-M (Right side)		Pre-M (Left side)	
	C1	C2	C1	C2
Yes	25	01	25	01
No	04	00	04	00
	*(X_2d.f_ =0.36,p>0.05)*		*(X_2d.f_ =0.36,p>0.05)*	

**Table 7:** Association of periodontitis and MCI in post-M group (intra-group analysis)

Periodontitis	Post-M (Right side)			Post-M (Left side)		
	C1	C2	C3	C1	C2	C3
Yes	03	20	03	03	20	03
No	00	04	00	01	03	00
	(*X*_2d.f_ =*1.15,p>0.05)*			(*X*_2d.f_=*1.04, p>0.05)*		

## DISCUSSION

Bone mass decreases with age and is irrespective of gender. The rate of bone loss in healthy men is low, in the order of 3-5% per decade, whereas in women the process is more complicated.[23] The rate of bone loss in the initial 10 post-menopausal years varies widely. It ranges from less than 1% to more than 5% per year for cancellous bone and from 0.5 to 2% for cortical bone. Thereafter post-menopausal bone loss declines in rate.[23] Early menopause, either naturally occurring, drug or surgically induced, without hormone replacement therapy predisposes to osteoporosis.[24] The earliest suggestion of an association between osteoporosis and oral bone loss was made in 1960.[25] The mandible seems to undergo a decrease in mineralized bone throughout life, as with other bones. Hildebolt,[26] in a recent review of the literature, states that an association exists between osteoporosis and oral bone loss and mandibular morphology alters due to osteoporosis.[27]

A large fraction of the adult population sees dentist regularly and panoramic radiographs of jaw bones are often made at these visits. The most commonly studied measures of mandibular morphology on DPRs in relation to osteoporosis include thickness and integrity of inferior border (endosteal and intracortical resorption, respectively)[27] and some radiomorphometric indices which can be utilized as tools in detection of low BMD. The goal of such screening is not to diagnose osteoporosis but to identify the risk for bone mass loss and appropriately refer the patient for assessment by bone densitometry.[28]

Patients in our study groups were not selected on the basis of any radiographic or medical criteria, which would define an individual as ‘normal’ or ‘osteoporotic’. Based on medical history patients on medications or suffering from systemic diseases affecting BMD were excluded from the study. The study group, therefore, represents patients undergoing normal physiologic bone changes with age who had undergone DPR examination as a part of their routine dental treatment.

MCI is a qualitative index of cortical morphology. Results of studies[18,29] suggest that MCI classification based on panoramic radiographs may be a useful index for the diagnosis of osteoporosis. It has been shown that mean mandibular BMD assessed by DXA has a significant relationship with the MCI scale with mandibles classified as C3 having the lowest BMD.[10] Additionally, the role of clinical dentists in the identification of mandibular cortical erosion detected at panoramic radiographs of postmenopausal women results in 73% of correctness in the identification of low mineral bone density.[30] To our knowledge, this is the first study in which the groups were divided based on the menopausal status where the pre menopausal group included patients ranging 22-45 years and post menopausal group included patients from 45-85 years. In this study, MCI appearance was related to the menopausal status of the patients suggesting that onset of menopause leads to changes in mandibular cortical morphology. C1 was most common in pre menopausal group. Individuals with C3 appearance was seen in post-menopausal group only after the age of 54 years and an age related increase in number of patients with C3 appearance was also observed, thus reflecting age related changes. Concurrently, the present study is in agreement with the results of Knezovic-Zlataric *et al*,[31] reflecting age related changes.

Hence, in our study, MCI appearance was related to the menopausal status and also the age of the patients.

Considerable attention has been paid to low skeletal bone mass screening based on cortical width measurements at the mental foramen area.[32] Recently, Dutra *et al*.[33] showed that the measurement of MI is accurate in panoramic radiographs and representative of the true bone status. Most authors[32,34] suggest that patients with the thinnest mandibular cortices (≤3 mm) should be referred for further osteoporosis investigation because it is this group that has the highest likelihood of osteoporosis. In our study mean values of pre-menopausal group were higher than the post-menopausal, also both groups showed statistically significant differences. Results of MI showed a negative correlation with age i.e. decreasing mean values with increasing age. However, the mean values reported in our study were higher than those reported in previous studies,[35,36] which can be explained on the basis of ethnic background as to best of our knowledge this is the first study done in Indian population.

PMI is a radiomorphometric method presented in 1991 by Benson *et al*.[19] It is partly based on the Wical and Swoope[37] method, which suggests a relation between residual ridge resorption and mandibular height below the inferior edge of the mental foramen. They suggest that despite the alveolar bone resorption above the foramen, the distance from the foramen to the inferior border of the mandible remains relatively constant throughout life. The distance below the foramen in a non-resorbed mandible is approximately one third of the total height of the mandible in that region.[37] Thus, the PMI provides a measure of mandibular cortical thickness for normal mandibular size and it could be used for the evaluation of local bone loss in dental practice.[38]

Our study reveals higher mean values in pre-menopausal group than post-menopausal group with statistically significant differences between two groups. At the same time mean values in post menopausal group were similar to those reported by Klemetti *et al*,[18] in a study of Finnish post menopausal population. When correlated with age a positive correlation was seen in both the groups. However, after dividing the post-menopausal group based on age, sub-group with age more than 60 years showed a negative correlation with age. These results are consistent with the results of Taguchi *et al*,[39] who suggested that in females, PMI demonstrated a gradual increase until sixth decade and then decreased.

In recent years, association of periodontal disease and osteoporosis has been extensively studied. Kribbs[40] showed no significant differences in periodontal measurements between osteoporotic and normal individuals. In contrast, other authors have found a significant relation between systemic osteoporosis and loss of periodontal tissue.[41,42] Our study did not find any association between periodontal status and MCI appearance. These results are consistent with the results of other studies[43] which suggest that osteoporotic changes after menopause are not essential factors in causing periodontal bone loss, but may influence the speed of bone loss.[44]

This study is different from studies done in past as the study groups in those have been divided on either age basis or MCI status as seen on radiographs and none of studies have excluded various systemic, drug or surgical factors that may affect the bone mineral density in general and of mandible.

The limitation of our study may be its smaller sample size and that it presents observations made by a single observer; as the variations in measurements may prove to be a serious drawback in practical clinical use of the indices, particularly if use by general dental practitioners is foreseen.[9,10,22]

In conclusion our study demonstrates significant differences between the pre-menopausal and post menopausal groups for MI and PMI. These indices, after a little training, can help the clinician in early screening of patients at high risk of osteoporosis and thus enable a dentist to help his patients by referring them for further investigations of bone density measurements.
